# Optical control of carrier-mediated ion transport by photoswitchable lipids

**DOI:** 10.1039/d5nr04234h

**Published:** 2025-12-04

**Authors:** Juergen Pfeffermann, Rohit Yadav, Toma Glasnov, Oliver Thorn-Seshold, Peter Pohl

**Affiliations:** a Institute of Biophysics, Johannes Kepler University Linz Linz Austria peter.pohl@jku.at; b Institute of Chemistry, University of Graz Graz Austria; c Faculty of Chemistry and Food Chemistry, Dresden University of Technology Dresden Germany

## Abstract

We report a molecular strategy for precise, reversible, and noninvasive photoregulation of ion-selective membrane transport. Embedding azobenzene-containing photolipids into bilayers enables nanoscale control over the interaction and mobility of small-molecule ion carriers. Photoisomerization alone produces only minor changes in baseline conductance, consistent with the limited influence of small bilayer thickness variations on ion permeability, yet it elicits striking responses in the presence of mobile carriers. A newly designed protonophore exhibits proton-selective currents that increase by up to 200-fold under UV illumination and revert to baseline within milliseconds upon blue light. These effects cannot be explained by thickness or fluidity changes. Instead, they arise from light-dependent interactions between azobenzene moieties and the carrier that increase the membrane-bound carrier concentration and lower the effective barrier for transbilayer permeation *via* interfacial dipole and packing modulation. Because this mechanism relies entirely on chemical design – without genetic modification – and is compatible with photoswitches operating at longer wavelengths, it establishes a versatile framework for dynamic, light-driven control of ion transport in biological membranes and synthetic nanosystems.

## Abstract 2

We report a molecular strategy for precise, reversible, and noninvasive photoregulation of ion-selective membrane transport.

## Graphical abstract

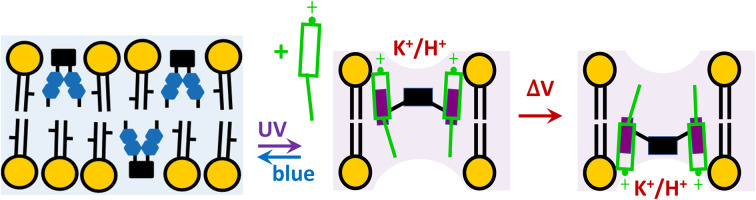

## Introduction

The regulated transport of ions across biological membranes is central to cellular function. In excitable cells, rapid changes in sodium and potassium permeability enable neuronal signaling,^1^ while in energy metabolism, regulated proton flux sustains ATP synthesis and limits reactive oxygen species generation.^2,3^ Achieving precise and reversible control of ion-selective transport by external stimuli is a key goal in both fundamental biophysics and applied nanomaterials research.

**Fig. 1:**
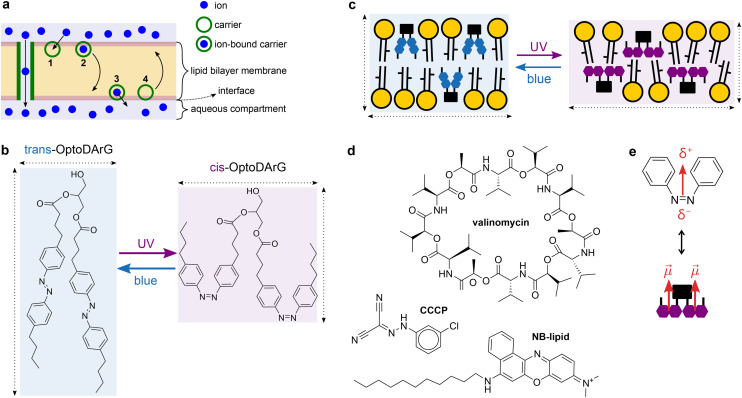
Components and concepts underlying photolipid-regulated nanoscale carrier transport. (a) Channels and nanoscale carriers facilitate transmembrane ion transport across biological membranes along the electrochemical gradient.^8^ Carrier transport typically occurs in four steps: 1, an ion at the bilayer–aqueous interface associates with an interfacially-adsorbed carrier molecule; 2, the carrier–ion complex traverses the membrane; 3, the ion dissociates from the carrier and is released into the membrane's aqueous surroundings; 4, the free carrier can traverse the membrane as well; it is free to bind another ion or recycle to the original side. (b) The photolipid OptoDArG in its *trans* and *cis* state: blue light (488 nm) generates mainly the former, UV light (375 nm) generates mainly the latter. For brevity, the photoequilibria will henceforth be indicated as simply “*trans*” (blue light) and “*cis*” (UV light). *cis*-OptoDArG is broader and shorter than *trans*-OptoDArG. (c) Both photoisomers incorporate into lipid bilayers and cellular membranes. Photoisomerization of membrane-embedded photolipids causes changes in global material properties owing to changes in molecular structure (*cf*. panel b). Structurally, photoisomerization to *cis*-OptoDArG by UV light increases bilayer surface area but reduces bilayer thickness. (d) Chemical structures of the carriers used in this study: the cationic K^+^ ionophore valinomycin, the anionic protonophore CCCP and NB-lipid, a lipidated Nile Blue derivative which acts as a cationic protonophore. (e) *cis*-azobenzene has a dipole moment, 
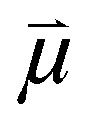
 of magnitude 3 D; *δ*^−^ and *δ*^+^ indicate negative and positive partial charge. A simplified view of the expected orientation of *cis*-OptoDArG in the lipid bilayer (*cf*. panel c) indicates that, on average, the azobenzene moieties’ dipole moments point towards the interfaces.

Light is particularly attractive because it affords site-specific, minimally invasive, temporally precise, and reversible control over membrane conductance and permeability.^4–6^ The membrane permeability coefficient *P* relates steady-state ion flux *J* to the transmembrane concentration gradient Δ*c* as *J* = −*P*·Δ*c*.^7^ Classical theory (Fig. 1a) established that small changes in bilayer thickness or ion pairing have limited impact on *P*, whereas nanoscale carriers and channels can dramatically enhance ion translocation by providing polar pathways and reducing the Born energy penalty.^8,9^

Here, we show that nanoscale photolipid dopants couple light-induced conformational changes to carrier-mediated ion transport. Beyond the established thickness modulation from azobenzene photoisomerization, photolipid incorporation produces pronounced changes in carrier-induced conductivity by: (i) modifying interfacial packing, (ii) creating conformation-dependent interfacial binding sites for the carrier, and (iii) lowering the effective barrier for flip-flop and transbilayer passage *via* interfacial dipole potential and packing modulation. This establishes a molecular platform for reversible, optical switching of ion transport across synthetic and biological membranes, compatible with photoswitches operating at longer wavelengths.^6,10,11^

### Regulation of valinomycin–K^+^ flux across photoswitchable bilayers

Our approach leverages previous findings that the photolipid OptoDArG (Fig. 1b) spontaneously incorporates into lipid bilayers and can undergo rapid isomerization there by exposure to intense UV (375 nm) or blue (488 nm) light (irradiance of several hundred W cm^−2^).^12^ Consequent changes in molecular structure alter the material properties of the embedding bilayer membrane at the millisecond scale (Fig. 1c).^12,13^ We conducted initial experiments with the electrically neutral dodecadepsipeptide macrocycle valinomycin (Fig. 1d) synthesized by *Streptomyces fulvissimus*.^14^ Valinomycin can traverse the membrane both as a neutral molecule without K^+^ and as a valinomycin–K^+^ complex,^9,15^ thus acting as an established K^+^-selective carrier (Fig. 1a).^9,15^

**Fig. 2:**
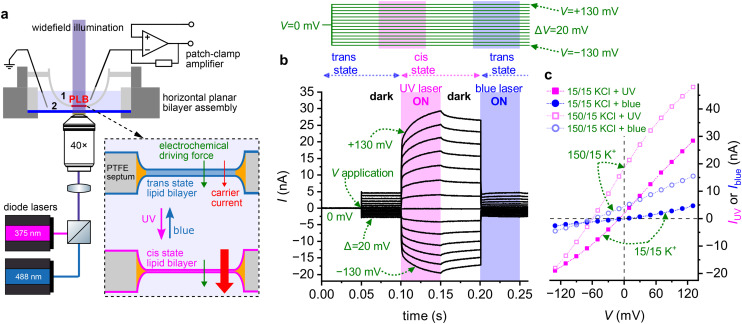
Photoisomerization of photoswitchable PLBs regulates the flux of valinomycin–K^+^ across the membrane. (a) Schematic of the measurement setup. The patch-clamp amplifier was used to clamp a transmembrane potential, *V*, across the horizontal PLB and measure the ensuing current, *I*. A blue and UV laser were used to rapidly isomerize the photoswitchable PLB. The resulting changes in carrier current at constant electrochemical driving force are measured. Side 1 and side 2 are indicated. (b) Voltage–clamp current, *I*, recordings on a photoswitchable planar lipid bilayer (PLB) with 10 µM valinomycin added to the aqueous compartments (15 mM KCl, 10 mM HEPES pH 7.4) on both sides. Voltage protocol (inset above): between 0 and 50 ms, *V* = 0 mV and between 50 and 300 ms, *V* = 130 mV to −130 mV with 20 mV steps between separate sweeps; the delay between consecutively recorded sweeps was 1 s. As indicated, the PLB was exposed to UV light between 100 and 150 ms (magenta bar) and to blue light between 200 and 250 ms (blue bar). UV light evokes a rapid increase in *I* that is effectively abrogated by blue light exposure. (c) *I*–*V* curves constructed from current traces recorded as in panel b by averaging *I* within certain time intervals and plotting the obtained values over *V*. *I*_UV_ values were calculated by averaging *I* between 125 and 150 ms (magenta squares); *I*_blue_ values by averaging *I* between 225 and 250 ms (blue circles). *I*–*V* curves are given for symmetric (15 mM KCl at both sides, closed symbols) and asymmetric ionic conditions (150 mM KCl at side 1, 15 mM KCl at side 2, open symbols). The K^+^ selectivity is retained upon UV light exposure.

Using voltage-clamp measurements on photolipid-containing planar lipid bilayers (PLBs; folded from 80 wt% *E. coli* polar lipid extract (PLE) with 20 wt% OptoDArG; schematic of the experimental setup in Fig. 2a) in the presence of 10 µM valinomycin, we observed an increase in current, *I*, within milliseconds of UV light exposure (Fig. 2b). To calculate membrane conductance *g*_0_ at *V* = 0 mV, we fitted the equation *I*(*V*) = *g*_0_*·*(1 + *αV*^2^)*·V* + *o* to the current–voltage (*I*–*V*) curves recorded under symmetric conditions (Fig. 2c) where *I*(*V*) is the current at a particular voltage, *g*_0_ the conductance at *V* = 0 mV and *α* is a supralinearity factor;^7^*o* accounts for a small current offset. *g*_0_ was sensitive to illumination. The ratio *g*_0,UV_/*g*_0,blue_ was 7.5 ± 1.1 (mean ± SEM, *N* = 4), where *g*_0,UV_ denotes the conductivity at 0 mV following photoisomerization to the photostationary *cis* state induced by UV light and *g*_0,blue_ the conductivity at 0 mV following photoisomerization back to the photostationary *trans* state induced by blue light. Light sensitivity was conferred by OptoDArG, as indicated by the small value of *g*_0,UV_/*g*_0,blue_ = 1.2 (compare recordings of *I* in Fig. S2c), the latter likely reflecting minor changes in membrane temperature (a related observation in ref. 16).

*I*–*V* curves constructed from measurements under a 10-fold K^+^ concentration gradient (open symbols in Fig. 2c) resulted in reversal potentials, *V*_r_, of −56.5 mV (*trans* state) and −59.8 mV (*cis* state; both determined by interpolation), which is close to −59 mV anticipated from the Nernst equation. This shows that the selectivity of valinomycin for K^+^ was retained. We determined that, with variation of the UV laser power (Fig. S1b), the fit values for the rate of current increase linearly with irradiance (Fig. S1c), which is also true for the rate of capacitance change.^12^ The increase in *I* with UV light was clearly the result of photolipid photoisomerization, as evidenced by its abrogation by blue light (at 200 ms in Fig. 2b; rate ≈ 7000 s^−1^) which isomerizes the azobenzenes in OptoDArG's acyl chains back to the *trans* state. The UV-evoked increase in *I* does not spontaneously decay to the pre-UV-illumination level without exposure to blue light. This is emphasized by Fig. S1a in which UV light was applied but no blue light and *I* stayed high. Consequently, we conclude that heating or photodynamic effects did not play a determining role but that changes in bilayer properties that are associated with reversible photolipid photoisomerization did.

To account for the several-fold increment in *g*_0,UV_/*g*_0,blue_, we employ a modified Nernst-Planck equation in the small potential limit (compare eqn (72) in ref. 7):1
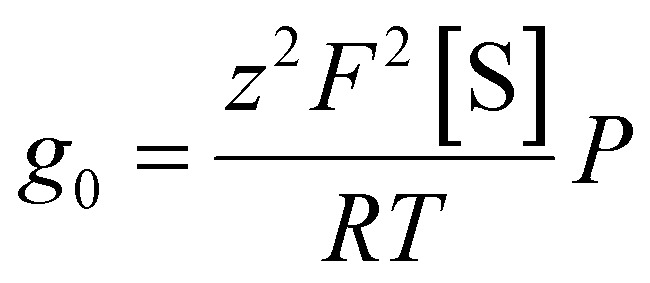
where *F*, *R*, and *T* denote the Faraday constant, universal gas constant, and absolute temperature, respectively. In the following, we restrict our analysis to monovalent ions, so the valence *z* is unity and will be omitted for clarity. Eqn (1) describes the functional dependence of *g*_0_ on *P*. For reasons outlined below, we assume a three-slab model of the lipid bilayer: the outer slabs represent the headgroup regions, the inner slab corresponds to the hydrophobic membrane core. Accordingly, eqn (1) describes the permeation through the inner slab, and [S] denotes the concentration of the charged species in the outer slabs – *i.e.*, after partitioning into the headgroup region, but with the charge still oriented toward the adjacent aqueous phase. In this model, *P* may be represented as *P* = *K*_p_·*D*/*d*, where *D* is the membrane diffusion coefficient, and *d* membrane thickness. The partition coefficient *K*_p_ which characterises the transfer of species *S* from one of the outer slabs into the inner slab (onto the top of the trapezoidal energy barrier), can be rewritten in terms of a Boltzmann distribution involving the free energy Δ*G* that opposes transmembrane movement. Substituting these expressions yields:2
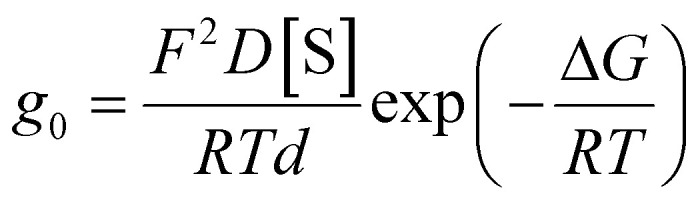


Eqn (2) does not allow to explain the roughly eightfold increase in *g*_0_ by alterations of *d* or *D* or a combination of both. *d* does not change by more than 10% upon photoisomerization, even if we attribute the entire 10% change in capacitance (Fig. S5b) to *d*. *D* is also unlikely to change strongly in fluid membranes – it has long been recognized that the diffusion coefficients contribute only marginally to membrane permeability differences, with partition coefficients playing the dominant role.^17^ Molecular dynamics simulations confirmed this finding for a weak base, even in cholesterol-containing membranes.^18^

Considering photoinduced changes in the dielectric constant, *ε*_hc_, and the resulting alterations in Δ*G* as a possible origin of the increase in *g*_0_ yields similarly unsatisfying results. *ε*_hc_ affects Δ*G* because electrostatic terms constitute the major contributions to Δ*G*. These include (i) ion self-hydration given by the Born energy, Δ*G*_b_, and (ii) interaction with the positive membrane dipole potential, *ϕ*_d_, giving rise to the dipole energy term, Δ*G*_d_, which is sensitive to the ion's sign.^19^ Remaining minor contributions include image forces and the energy required to insert the neutral species into the bilayer. To assess how strongly Δ*G* may be altered by changes in *ε*_hc_ we rely on two observations: (i) the electrostatic components of Δ*G* are inversely proportional to *ε*_hc_, and (ii) the Arrhenius activation energy *E*_v_ = 16 kcal mol^−1^ (ref. 20) being proportional to the unknown Δ*G* enables the use of Expression 2 to predict the expected tenfold change in g_0_ if illumination alters *ε*_hc_ by 10%. In our assessment, we attribute the entire increase in capacitance observed upon UV illumination to a rise in *ε*_hc_ from 2.1 to 2.3. As a result, the inversely proportional *E*_v_ is expected to decrease to approximately 14.6 kcal mol^−1^.

**Fig. 3:**
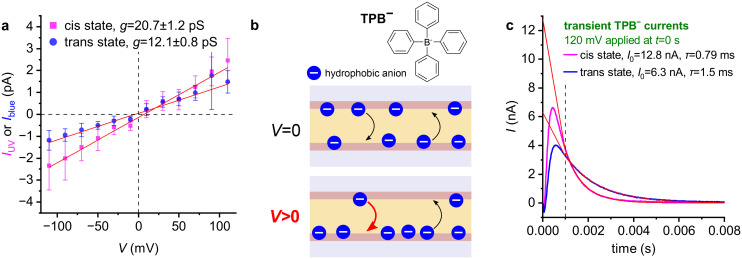
Photoisomerization has a comparatively small effect on background and hydrophobic ion conductance. (a) To infer the change in background ion conductance upon photoisomerization, recordings as in Fig. 2b but in the absence of carrier were conducted; the buffer was 15 mM KCl, 10 mM HEPES pH 7.4. *I*–*V* curves were constructed from the obtained records, as described in Fig. 2c, and data points from 5 separately prepared experiments were averaged (error bars correspond to SD). The curves were fit by linear models with offset, with each point weighted by 1/SD^2^ (*R*^2^ of 0.97 [*cis* state] and 0.96 [*trans* state]). Background ion conductance increases by ≈71% in the bilayer *cis* state, a small effect compared to the experiments with valinomycin and CCCP. (b) Tetraphenylborate (TPB^−^) is a hydrophobic anion that permeates the membrane. At low bulk TPB^−^ concentrations, voltage application leads to the redistribution of membrane-adsorbed ions between the leaflets without considerable contribution from the bulk.^23^ Hence, in contrast to carriers, chemical uptake and release reactions at the interface play little role (*cf*. Fig. 1a). (c) Transient TPB^−^ currents evoked upon the application of *V* = 120 mV at *t* = 0 s with the bilayer in the *cis* (magenta lines) or *trans* state (blue lines); TPB^−^ was 200 nM in 100 mM NaCl, 10 mM HEPES pH 7.4 (conditions similar to ref. 23). Each transient was recorded twice with a delay of 800 ms to check for steady-state conditions: the curves overlap perfectly. Due to a 2 MΩ resistance in series with the PLB, necessary for capacitance compensation, and the deployed 10 kHz Bessel filter, the transients are smoothed. Hence, to estimate initial current *I*_0_ and decay time *τ*, data points from 1 to 8 ms were fit with a monoexponential model. The fits (red lines) indicate a modest effect (around 2-fold) of bilayer state on *I*_0_ and *τ*, which is small compared to the experiments with valinomycin and CCCP.

Yet, even such modest changes in *ε*_hc_ should be ruled out, as the permeability to other ions did not show similar changes. Since their conductance is also sensitive to changes in *ε*_hc_,^21^ we would have expected larger differences in *g*_0_ between the *trans* and *cis* bilayer states for inorganic ions such as Cl^−^ and for organic anions like tetraphenylborate (TPB^−^). Specifically, literature reports suggest Δ*G* ≈ 23.6 kcal mol^−1^ for Cl^−^ permeation.^22^ Using the same logic as above, a 10% increase in *ε*_hc_ would lower Δ*G* to ≈21.5 kcal mol^−1^ and, *via* Expression 2, increase *g*_0_ by ≈35-fold. However, photoisomerization of OptoDArG led to only a modest increase in the background Cl^−^ conductance – from 12.1 ± 0.8 pS in the *trans* state to 20.7 ± 0.8 pS in the *cis* state (Fig. 3a). Although the resulting 71% increase was notable, it remained several-fold smaller than the ≈35-fold increase predicted by changes in *ε*_hc_, reinforcing that photoisomerization-evoked changes in dielectric constant are not the principal driver of the observed conductance changes with valinomycin–K^+^ complexes.

**Fig. 4:**
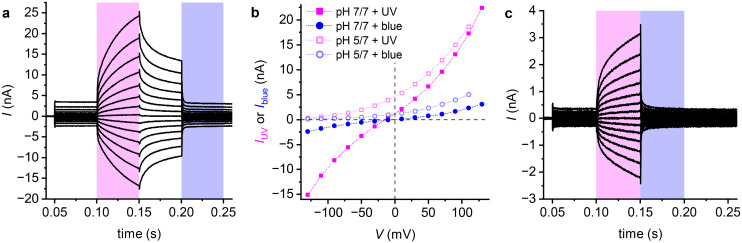
The transmembrane flux of the anionic protonophore CCCP^−^ is rapidly modulated by photoisomerization of membrane-embedded photolipids. (a) Voltage–clamp current recordings on a photoswitchable PLB with 10 µM CCCP added to the aqueous compartments (100 mM KCl, 20 mM HEPES pH 7.0) on both sides. The recordings were made as in Fig. 2b. As with valinomycin–K^+^, *I* increases upon UV light exposure and blue light reverts this increment. That is, the flux of CCCP^−^ is increased across the *cis* state membrane. (b) *I*–*V* curves constructed as described in Fig. 2c for recordings under symmetric (pH 7 at both sides; closed symbols) and asymmetric conditions (pH 5 at side 1, pH 7 at side 2; open symbols). Upon creating the gradient in pH by the addition of HCl to compartment 1, *V*_r_ shifted towards large negative values, consistent with proton-selective transport. Whilst exposure to UV light increased *I*, it remained selective for H^+^. (c) Photoswitchable PLB with 2.5 µM CCCP added to the aqueous compartments (15 mM KCl, 10 mM HEPES pH 7.4) on both sides. Voltage protocol as in panel a. In contrast to panel a, UV light exposure between 100 and 150 ms was immediately followed by blue light from 150 to 200 ms. This record shows that blue light leads to the immediate abrogation of the UV-evoked increment in *I* as a result of switching back to the *trans* bilayer state.

Second, we assessed the photoeffects on TPB^−^-mediated conductivity. Its membrane permeability is much higher than that of inorganic ions.^23,24^ It is so large that the diffusion of TPB^−^ towards the membrane limits the steady-state current. At the TPB^−^ concentrations used (200 nM), application of a voltage (*V* = 120 mV at *t* = 0 ms in Fig. 4c) leads to the redistribution of membrane-adsorbed TPB^−^ between the adsorption sites in each leaflet (schematic in Fig. 4b), which results in an exponentially decaying current.^23^ A monoexponential fit to the transient currents in Fig. 4c allowed us to estimate the initial current, *I*_0_, and time constant of the transient, *τ*.^24^ Both parameters increase by a factor of ≈2 in the *cis* state membrane (inset in Fig. 4c). This indicates that the flux between the adsorption sites increases in the presence of *cis*-OptoDArG, but the increase is small compared to the effects on valinomycin–K^+^. From these observations, we conclude that *ε*_hc_-mediated flux amplifications cannot govern the photoeffect on valinomycin–K^+^ currents.

With changes in *ε*_hc_ excluded as the source of the photoinduced modulation of Δ*G* (Expression 2), we considered an alternative explanation: variations in the membrane dipole potential, *ϕ*_d_, leading to changes in Δ*G*_b_. Primarily generated by phospholipid carbonyl groups and ordered interfacial water, *ϕ*_d_ (typically around +250 mV inside the membrane) opposes cation partitioning and favors anion partitioning.^25,26^ Permeability differences between structurally similar organic cations and anions can span up to seven orders of magnitude.^25^ A decrease in *ϕ*_d_ by several tens of millivolts could plausibly increase valinomycin–K^+^ permeation by an order of magnitude.^27^ Such a reduction might arise from (i) dipole moment differences between *cis*- and *trans*-azobenzenes in OptoDArG (Fig. 1e),^28^ (ii) looser lipid packing due to the larger footprint of *cis*-OptoDArG (Fig. 1c), or (iii) altered orientation or density of interfacial water.^29^ However, our TPB^−^ data argue against this mechanism. While reduced *ϕ*_d_ would align with increased valinomycin conductance, it cannot account for the observed doubling of TPB^−^ conductance; in fact, it should have decreased. These findings thus rule out *ϕ*_d_ changes as the main driver of the ≈10-fold increase in *g*_0_ for valinomycin–K^+^ upon UV-induced *cis*-OptoDArG formation (Fig. 3).

As Δ*G* does not explain the increase in *g*_0_ and changes in *D* or *d* were excluded previously, [S] remains the only plausible factor in eqn (1). The much larger current increase for valinomycin–K^+^ compared to TPB^−^ suggests a mechanistic distinction: valinomycin reversibly binds K^+^ at the membrane interface. This process is governed by (i) association and dissociation rate constants *k*_A_ and *k*_D_, respectively, with their ratio *K*_A_ = *k*_A_/*k*_D_ defining the equilibrium constant, and (ii) valinomycin partitioning from the aqueous solution into the membrane. In the absence of photolipids, both *K*_A_ and valinomycin partitioning were found to depend on acyl chain length and unsaturation.^30^*K*_A_ increased from 1.5 in membranes with monounsaturated oleoyl (C18:1) chains to 9 with polyunsaturated linolenoyl (C18:3) chains. Since the *trans*–*cis* isomerisation of the photolipid mimics the changes in headgroup spacing observed with increasing unsaturation, we expect a higher *K*_A_ in *cis* bilayers. As a consequence, [S] increases and thus *I*, even when translocation remains rate-limiting. In other words, the resulting accumulation of valinomycin–K^+^ complexes enhances transmembrane K^+^ flux. The observed increase in valinomycin–K^+^ current through *cis*-state PLBs closely matches the previously reported sixfold rise in *K*_A_,^30^ lending credibility to this mechanism.

### Regulation of CCCP flux across photoswitchable bilayers

The mechanism identified above for valinomycin suggests that chiefly those ionophores or protonophores for which an increase in *K*_A_ can be expected should be affected by the photolipid's conformational change. This likely excludes the protonophore carbonyl cyanide *m*-chlorophenylhydrazone (CCCP), as it is well established that CCCP protonation is diffusion-limited,^31^ effectively disavowing a phototriggered increase in the equilibrium constant *K*_A_ and the surface p*K*_a_.

We were therefore surprised to observe a similar increase in conductance after replacing valinomycin with CCCP. *g*_0_ increased under UV illumination (Fig. 4a) by a factor 7.4 ± 0.3 (mean ± SEM of *N* = 3), closely resembling the behavior of valinomycin–K^+^. Again, ion-selectivity was preserved, as indicated by the sustained large negative *V*_r_ under a nominal 2 unit pH gradient (Fig. 4b). As in the case of valinomycin, *P* was swiftly modulated by light: triggering the blue laser immediately abolished the UV-induced increase in *I*, reflecting a swift return to the membrane's *trans* state (Fig. 4c).

As established for valinomycin, an increase in [S] is the most plausible explanation for the enhancement of *g*_0_. Since a shift in *K*_A_ is unlikely, we propose that the photolipids introduce binding sites for CCCP^−^, with fewer sites available in the *trans* than in the *cis* state. This hypothesis can be tested by measuring the surface potential, *ϕ*_s_. While absolute *ϕ*_s_ measurements are difficult to perform on planar lipid membranes, measurements of transmembrane differences in *ϕ*_s_, Δ*ϕ*_s_, are feasible. These rely on the dependence of membrane capacitance on the difference in boundary potentials, Δ*ϕ*_b_, across the bilayer, where Δ*ϕ*_b_ = Δ*ϕ*_s_ + Δ*ϕ*_d_ and Δ*ϕ*_d_ is the dipole potential difference between the two membrane–water interfaces.

To introduce asymmetry in leaflet composition, we replaced symmetric OptoDArG with 20 wt% 1-stearoyl-2-oxy-4-[4-(4-butylphenylazo)phenyl]butanoyl-*sn-glycero*-3-phosphocholine (OxyAzoPC) in only one leaflet. Importantly, due to its zwitterionic headgroup, OxyAzoPC does not undergo flip-flop. Measurements revealed that switching from *trans* to *cis* decreased Δ*ϕ*_b_ by ≈6 mV in the presence of CCCP (Fig. S6). Assuming Δ*ϕ*_d_ remains unchanged, this suggests greater CCCP^−^ binding to azobenzene groups in the *cis* state, consistent with our hypothesis.

To relate the photolipid-induced increase in surface CCCP concentration to its membrane adsorption in the absence of photolipid, we conducted experiments under a pH gradient. We made the following assumptions: (i) protonation/deprotonation reactions at the interface are at equilibrium, as they are faster than membrane transport, (ii) the membrane affinity for charged and neutral forms is equal, since the anion's negative charge remains near the interface,^31^ and (iii) the p*K*_a_ of 6.1 is the same in bulk and at the surface.^31^ At pH 5.0, ≈7% of CCCP is charged; at pH 7.4, ≈95% is charged. Subtracting the 6 mV Δ*ϕ*_s_ difference (from the difference in Δ*ϕ*_b_ due to CCCP adsorption to the *trans* and *cis* state membrane in the absence of a pH gradient) from the 10 mV Δ*ϕ*_s_ difference observed across a bilayer with one leaflet at pH 5 and the OxyAzoPC-containing leaflet at pH 7.4 leaves 4 mV. These 4 mV represent ≈90% of CCCP molecules adsorbing without photolipid.

Assuming that *ϕ*_s_ scales with the interfacial concentration of CCCP^−^, we estimate that 20 wt% *cis*-OxyAzoPC (which carries one azobenzene moiety) alone binds ≈1.5 times more CCCP^−^ than a control bilayer lacking photolipid. Accordingly, 20 wt% *cis*-OptoDArG (with two azobenzene moieties per molecule) may be expected to bind three times more CCCP^−^ than the bilayer alone. Together, both lipid-bound and OptoDArG-bound CCCP^−^ result in four times an abundance of binding sites compared to control conditions. The observed 7.4-fold light-induced increase in *g*_0_ exceeds this value, suggesting that not only binding but also the transport rate may have been increased. One possibility is that OptoDArG–CCCP^−^ complexes contribute directly to charge transport. The 4.3-fold increase in molecular mass upon complex formation would reduce Δ*G*_b_ by nearly a factor of two – an effect that together with the augmented binding site density would be consistent with the observed rise in *g*_0_.

### Strong photoeffects on proton-selective currents

To maximize the light effect and improve suitability for *in vivo* studies, we aimed to reduce the dark current (*i.e.*, the current when the photolipid is in its *trans* state). To this end, we replaced the anionic protonophore CCCP with a cationic protonophore – a weak base that crosses the membrane in its protonated positively-charged form. The rationale is that cationic protonophores have a higher Δ*G* than anionic ones due to the positive values of *ϕ*_d_. We selected Nile Blue (NB), a red fluorescent benzophenoxazine dye previously described as a mitochondrial uncoupler.^32^ Since NB is relatively inefficient as a protonophore, we increased its hydrophobicity by attaching a lipid chain, generating NB-lipid (Fig. 1d). Upon adding NB-lipid to both sides of an OptoDArG-containing membrane, we observed gradual insertion into the bilayer, accompanied by a corresponding increase in membrane conductance, *g*_0_ (Fig. S3). The recorded *I*–*V* curves exhibited a characteristic supralinear shape, consistent with the trapezoidal profile of Δ*G*.^7^

**Fig. 5:**
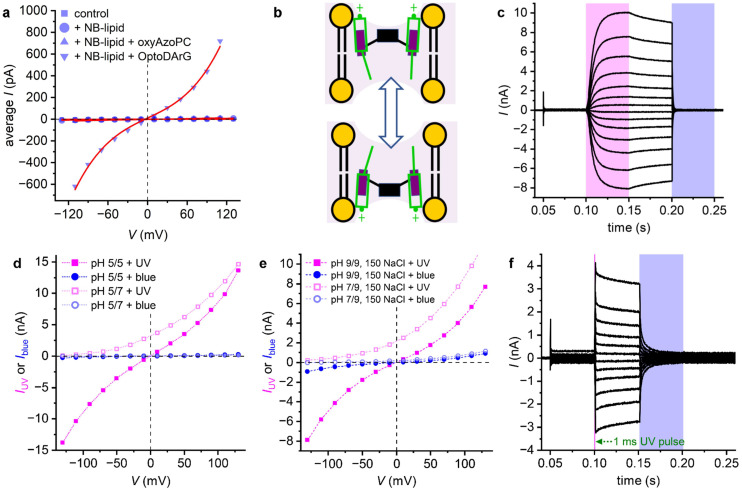
OptoDArG significantly augments the protonophoric activity of NB-lipid and allows its photoregulation. (a) Representative *I*–*V* curves recorded on PLBs folded from pure *E. coli* PLE (no NB-lipid), 99 wt% *E. coli* PLE with 1 wt% NB-lipid (+NB-lipid), 89 wt% *E. coli* PLE with 10 wt% OxyAzoPC and 1 wt% NB-lipid (+NB-lipid + OxyAzoPC), 79 wt% *E. coli* PLE, 20 wt% OptoDArG and 1 wt% NB-lipid (+NB-lipid + OptoDArG). (b) *g*_0_ was significantly increased only in the presence of both OptoDArG (glycerol and lipid backbone in black, azobenzene moieties in purple) and NB-lipid (green), indicating a decrease in Δ*G*. No flip-flop is observed when OptoDArG is substituted for OxyAzoPC. (c) Voltage–clamp current recordings with PLBs containing 20 wt% OptoDArG and 1 wt% NB-lipid. The solution contained 15 mM KCl, 10 mM HEPES pH 7.4. Recordings were made as in Fig. 2b. In this record, exceptionally high on–off current regulation by light (*I*_UV_/*I*_blue_ > 200) was achieved. (d) *I*–*V* curves constructed from current records as described in Fig. 2c. Conditions were symmetric (100 mM KCl, 20 mM MES, pH 5 at both sides; closed symbols) and asymmetric (pH at side 2 increased to 7; open symbols). NB-lipid currents are proton-selective. (e) Even at physiological salt concentrations (150 mM NaCl, 20 mM TRIS, pH 9), H^+^-selectivity is retained. This can be appreciated from the large negative-going shift in *V*_r_ upon reducing pH in compartment 1 by the addition of acid. (f) Recordings made as in panel c whereby the duration of UV light exposure was reduced to 1 ms (*V* ranged from 110 mV to −110 mV). Even short pulses of UV light can effectively regulate membrane proton permeability.

Importantly, the relatively large currents in Fig. S3 were recorded in membranes containing OptoDArG. In its absence, currents were much smaller (Fig. 5a), consistent with a large Δ*G*. Unlike in the CCCP experiments, the presence of OptoDArG was evident even when the bilayer was in the *trans* state. To clarify whether the increased current was due to additional binding sites provided by the photolipid, we replaced OptoDArG with OxyAzoPC and monitored *g*_0_. Importantly, *g*_0_ remained nearly unchanged with OxyAzoPC (Fig. 5a), suggesting that the flip-flop capability of OptoDArG^33,34^ is essential for the conductance increase. These observations can be explained by the formation of a nanoscale complex between OptoDArG and NB-lipid, which flips across the membrane and mediates proton transport (Fig. 5b).

Upon UV illumination of OptoDArG and NB-lipid-containing membranes, we observed a >200-fold increase in *g*_0_ and thus H^+^ permeability (Fig. 5c). Despite notable variability across replicates (*e.g.*, freshly prepared chambers and different lipid mixtures; Fig. S5a), the dark current remained generally low, and the light-induced increase consistently high (*g*_0_ = 4.8 ± 2.1 nS, fold increase in *g*_0_ with UV light: 85 ± 38, mean ± SEM of *N* = 5).

The increase in *g*_0_ upon UV exposure is, at least in part, due to new NB-lipid binding sites exposed by azobenzene moieties. Supporting evidence comes from the change in Δ*ϕ*_b_ in membranes with OxyAzoPC in only one leaflet (Fig. S6). Since OxyAzoPC does not flip-flop, Δ*ϕ*_b_ remains constant over time. Despite more carrier molecules, no significant increase in *g*_0_ is observed (Fig. 5a). In contrast, OptoDArG induces a rise in *g*_0_, suggesting that both binding partners – the azobenzene moiety and NB-lipid – must cross the membrane together. Only then does the increased interfacial concentration of protonated NB-lipid enhance *g*_0_ (Fig. 5c).

Experiments carried out with a transmembrane pH gradient confirmed that the current is proton-selective (Fig. 5d). Furthermore, this selectivity remained intact even in the presence of physiological salt concentrations (150 mM NaCl in Fig. 5e; 150 mM KCl in Fig. S4). Finally, even short UV light pulses (1 ms long) resulted in a sizable increment in *I* (Fig. 5f); this may be important for cell applications as it reduces the amount of energy delivered by light.

## Discussion

We demonstrated that isomerization of photoswitchable bilayers elicits effects beyond typical changes in bulk properties such as membrane thickness. This nanoscale conformational change photoregulates interfacial chemical reactions and, consequently, membrane transport. The substantial enhancements in carrier-mediated ion flux – up to two orders of magnitude – arise from increased interfacial concentrations of charged carriers and, in certain cases, additional transport rate amplifications mediated by light-responsive nanoscale complexes.

Interfacial concentration modulation is governed by (i) nanoscale changes in lipid packing due to photoisomerization and (ii) specific, conformation-dependent interactions between azobenzene moieties and carriers that form transient nanoscale complexes. For photolipid-enabled transport regulation, exploiting these phenomena is promising: photoisomerization increases the number of interfacial binding sites, notably those formed by *cis* azobenzene moieties. Recruitment of additional carrier molecules from bulk to membrane is evidenced by alteration in Δ*ϕ*_b_ upon switching OxyAzoPC from *trans* to *cis*. For OptoDArG, we observed a fourfold CCCP^−^ concentration increase. Together with the halving of Δ*G*_b_, this is largely consistent with the roughly sevenfold increase in CCCP-mediated *g*_0_ with *cis*-OptoDArG.

The larger increase in *g*_0_ with NB-lipid by up to two orders of magnitude cannot be explained by additional interfacial binding sites alone. OxyAzoPC provides binding sites yet does not substantially raise conductivity, indicating that specific organization and complexation are required. Robust proton-selective currents emerged only when OptoDArG was present alongside NB-lipid, implicating a second mechanism: enhanced proton transport *via* reduction in Δ*G*. We propose a mobile OptoDArG–NB-lipid complex that flip-flops across the bilayer, a process facilitated by *cis*-OptoDArG–induced thinning and reduced packing (Fig. 5b).

Two factors contribute to Δ*G* reduction: (i) the nanoscale 1 : 1 OptoDArG–NB-lipid complex increases the effective size of the transporting entity, reducing Δ*G*_b_ by about one third; (ii) NB-lipid lowers Δ*ϕ*_b_ (Fig. 5b). Therefore, NB-lipid dipoles align antiparallel to membrane dipoles (the positive charge points toward the aqueous solution), decreasing Δ*ϕ*_d_ sufficiently to offset the complex's effect on Δ*ϕ*_s_. Similar divergent effects on Δ*ϕ*_d_ and Δ*ϕ*_s_ are known for verapamil, whose positive charge also faces the aqueous phase while the negative pole points inward.^27^ The resulting decrease in Δ*ϕ*_b_ lowers Δ*G*_d_ and thus the overall Δ*G*.

Accordingly, the photoinduced increase in proton-selective current with OptoDArG and NB-lipid reflects the combined action of: (i) decreased lipid packing in the *cis* state, (ii) increased density of interfacial binding sites provided by *cis* azobenzenes, and (iii) a reduced Δ*G via* Born energy and dipole potential modulation.

Notably, the combination of NB-lipid and photolipids partially outperforms channelrhodopsins (ChR), an alternative method for generating light-switchable proton currents.^35–39^ At neutral pH, the photocurrents generated are comparable in magnitude (Fig. 5c) to those elicited by the human proton channel H_V_1 or ChR in transfected cells.^5,40^ As in the case of H_V_1 or ChR2, the current is highly selective for protons. In contrast to ChR2, where the ratio of proton to sodium permeability, *P*_H^+^_/*P*_Na^+^_, of 2 × 10^6^ to 6 × 10^6^ yields a substantial Na^+^ component at neutral pH in mammalian brain,^39^ NB-lipid–containing *cis*-state membranes exhibit negligible salt-ion contributions even at high salinity (Fig. 5e).

Our all-chemical, nanoscale photolipid platform enables millisecond-scale, light-dependent regulation of well-established, highly selective carriers, delivering large currents, submillisecond photoresponse, tunable ion scope, and a single-photon action spectrum extendable from UV/Vis to NIR/SWIR.^41,42^ Red-shifted azobenzene photoswitches and photolipids suitable for red and near-infrared actuation are already available.^43–46^ These attributes differentiate it from prior strategies: (i) photopharmacological control of drug conformation by light^6,10^ that typically use orthosteric or allosteric ligands to gate protein activity (*e.g.*, ion channels and other membrane transporters),^42,47^ (ii) molecular machines where ionophores are directly photosensitized^48^ where light-dependent currents are generated and ionophores *per se* are modified with light-sensitive moieties, (iii) rotary molecular motors which have been reported to increase membrane ion permeability upon irradiation,^49,50^ but have been shown to act at least partially through irreversible photodynamic membrane lipid peroxidation^51^ rather than drilling,^52^ (iv) artificial supramolecular channels,^53,54^ and (v) light-based approaches at regulating mechanosensitive, voltage-sensitive and other membrane-embedded channels by alterations in bilayer mechanical properties.^12,13,55–57^

Additionally, while the ChR-based optogenetic approach^35–39^ requires genetic transfection to achieve selective ion currents in response to incident light, our nanoscale chemical approach avoids this complication since both ion carriers and photolipids^12,58^ can be administered acutely *via* solution. This modular control complements protein-based optogenetics and should generalize to Ca^2+^, Na^+^, and K^+^ carriers, paralleling ChR engineering,^38,39^ but with faster, rational design cycles and broader spectral tunability.^41,42^

We envision photolipid-regulated carriers as a convenient and general nanoscale chemical toolkit for dynamic, light-based regulation of ion permeability in biological and synthetic cells, and in artificial neuronal systems. More broadly, photolipid-based optical control of interfacial nanostructure and reactivity should extend beyond ion transport, suggesting that the potential of these photoswitchable lipid reagents is greater than previously appreciated.

## Materials and methods

### Materials for planar lipid bilayer experiments

*E. coli* Polar Lipid Extract (PLE, item no. 100600) was obtained from Avanti Polar Lipids (distributed by Merck) and kept at −80 °C. OptoDArG was synthesized by the group of Dr Glasnov as described previously.^59^ Lipid aliquots and mixtures were prepared within amber glass micro reaction vials from lipids dissolved in chloroform. Prior to storage at −80 °C, solvent was evaporated by a mild vacuum gradient (Rotavapor, Büchi Labortechnik AG) and the dried lipids were flooded with argon. All aqueous buffers used in the PLB experiments were freshly prepared from laboratory-grade dry substances (supplied by VWR, Merck, or Fisher Scientific) dissolved in ultrapure water (>18 MΩ cm, Milli-Q water purification system) and pH-adjusted using a daily-calibrated pH meter (FiveEasy, Mettler Toledo). CCCP (carbonyl cyanide *m*-chlorophenylhydrazone, 98%, Thermo Scientific) and valinomycin were kept as stocks in DMSO. Sodium tetraphenylborate (>99.5%, T25402, Sigma-Aldrich) was added from a stock solution in ethanol. NB-lipid was synthesized in a parallel study, with synthesis and characterization given there as molecule **S43**.^42^ NB-lipid was kept in a dried state at −80 °C and aliquots were prepared in DMSO prior to measurement.

### Horizontal planar lipid bilayer experiments

Planar lipid bilayer (PLB) experiments with laser irradiation for rapid photoisomerization were conducted as recently described.^12^ A schematic of the setup is shown in Fig. 2a. Solvent-depleted horizontal PLBs (specific capacitance 0.75 µF cm^−2^) were folded from lipids spread on top of aqueous buffer in the lower and upper compartment of a custom-made chamber assembly made of PTFE.^60,61^

First, an aperture of around 70 µm in diameter in 25 µm-thick PTFE foil (Goodfellow GmbH) was created by high voltage discharge – this prepares the septum separating the macroscopic compartments. In this study, diameters were between 70 µm to 85 µm, with exceptions denoted explicitly. The PLB diameters given in the main text refer to the size of this aperture. After the septum was treated with 0.6 vol% hexadecane in hexane, hexane was allowed to evaporate for >1 h. The residual hexadecane facilitates the solvent annulus or torus that later laterally anchors the PLB within the aperture.^62^ The septum was attached by silicon paste to the lower side of the upper compartment of the chamber assembly. Lipids at the air–water interfaces were prepared by applying lipid mixtures dissolved in hexane at a concentration of 10 mg mL^−1^ onto both aqueous interfaces. After hexane had evaporated, a horizontal PLB was folded by rotation of the upper compartment of the chamber assembly.

A 30 mm-diameter cover glass (No. 1, Assistent, Hecht Glaswarenfabrik GmbH & Co KG) fixed with a threaded PTFE ring comprised the bottom of the lower compartment. The chamber assembly was installed on the sample stage of an Olympus IX83 inverted microscope equipped with an iXon 897 E EMCCD (Andor, Oxford Instruments Group). The chamber holder was equipped with screws for fine translation of the upper compartment in z direction to position the horizontal PLB within the working distance of a 40×/1.30 NA infinity-corrected plan fluorite oil immersion objective (UPLFLN40XO, Olympus) or a 40×/0.65 NA infinity-corrected plan achromat air objective (PLN40X, Olympus). The motorized microscope and real-time controller (U-RTC, Olympus) used for synchronizing lasers and electrophysiological acquisition were controlled using the proprietary cellSens software (Olympus).

For electrical measurements, a Ag/AgCl electrode with agar salt bridge containing 0.5 M KCl was put into each compartment and connected to the headstage of an EPC 9 patch-clamp amplifier (HEKA Elektronik, Harvard Bioscience). Headstage and chamber assembly were housed in a Faraday cage. Voltage-clamp measurements were conducted using PATCHMASTER 2x91 software (HEKA Elektronik, Harvard Biosciences). Current was analogously filtered at 10 kHz by a combination of Bessel filters and acquired at 50 kHz. Amplifier offsets were corrected by subtracting the average current recorded at *V* = 0 mV under symmetric conditions. Data recorded with PATCHMASTER was exported, analyzed, and graphed using Mathematica 14 (Wolfram Research) and OriginPro 2024 (OriginLab Corporation).

Rapid photoisomerization of photolipids embedded in horizontal PLBs was achieved by exposure to blue (488 nm diode laser, iBEAM-SMART-488-S-HP, TOPTICA Photonics) and UV laser light (375 nm diode laser, iBEAM-SMART-375-S, TOPTICA Photonics). Both lasers were digitally modulated and separately focused into the back-focal plane of the objective *via* the ZT488/640rpc main dichroic mirror (Chroma). The diameter of the blue laser profile at the sample stage was ≈58 µm (1/*e*^2^) whilst the UV laser profile spanned roughly 150–200 µm (its shape was less defined owing to the absence of a spatial filter). At a software-set output power of 200 mW (blue) and 70 mW (UV), ≈20–30 mW (blue and UV) exited the microscope objective depending on current alignment, determined by a photodiode (S120VC, Thorlabs). For convenience, we use the notation “*cis*” and “*trans*” to refer to PLBs containing mostly *cis* or mostly *trans* photolipids (corresponding to UV and blue light-evoked photostationary states).

### Vertical planar lipid bilayer experiments

Vertical PLBs were folded in a similar manner to the description above and as given in ref. 13. The Faraday cage was equipped with a magnetic stirrer positioned underneath the PTFE chamber, which allowed the aqueous compartments on both sides of the vertical PLB to be stirred by magnetic PTFE-coated stir bars. Ag/AgCl electrodes with agar salt bridges were used. The recordings were made with an EPC 10 patch-clamp amplifier (HEKA Elektronik, Harvard Bioscience).

### Biexponential function fits

To extract rate information from the time course of current upon light exposure, we fitted several sets of curves with the following bi-exponential equation:3
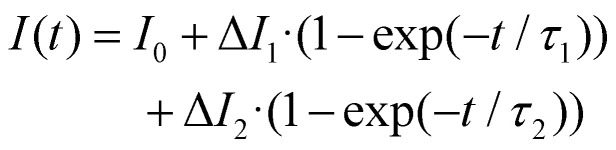
where *I*_0_ denotes basal current, Δ*I*_1_ and Δ*I*_2_ the increment in *I* associated with growth at rate 1/*τ*_1_ and 1/*τ*_2_. Typically, several curves were fit globally (Mathematica resource function MultiNonlinearModelFit) and curves recorded at lower voltages (|*V*| ≤ 30 mV) were neglected.

### Asymmetric PLBs and boundary potential measurements

Horizontal asymmetric PLBs were essentially folded as described above, with the distinction that the interfacial lipids applied from lipid mixtures in hexane differed between the two compartments of the chamber.^63^ Asymmetry here refers to a difference in the lipid composition of the two leaflets forming the bilayer.

The difference in boundary potential between the two sides of the membrane was determined by measuring membrane capacitance, *C*_m_, at different applied transmembrane voltages, *V*, using the EPC-9 patch-clamp amplifier. *C*_m_ was measured using the software lock-in amplifier implemented in PATCHMASTER (configuration: “Sine + DC” method with computer calibration, 20 mV peak amplitude, 833 Hz, 30 points per cycle, acquisition at 25 kHz). The DC voltage offset (*V*) was varied between −100 and +100 mV at 20 mV intervals. When the externally applied voltage cancels the intramembrane electric field – which corresponds to the difference in boundary potential of the two sides of the membrane – *C*_m_ is at its minimum.^64,65^ To infer this minimum from the parabolic dependence of membrane capacitance on *V*, the obtained *C*_m_–*V* data was fit by the following equation:*C*_m_(*V*) = *aV*^2^ + *bV* + *c*where *a*, *b*, and *c* are fit constants. The vertex of the parabola was calculated as –*b*/2*a*.

## Author contributions

JP, RY and PP conceptualized the research. RY conducted the experiments and with JP analyzed the obtained data. JP developed the methodology. JP and PP wrote the manuscript. TG synthesized OptoDArG. OTS synthesized NB-lipid and OxyAzoPC. PP acquired funding and supervised the research. All authors contributed to the editing of the manuscript.

## Conflicts of interest

The authors declare no competing interests.

## Supplementary Material

NR-018-D5NR04234H-s001

## Data Availability

All data supporting the findings of this study are included within the manuscript and its supplementary information (SI). Datasets have been uploaded to Zenodo at https://doi.org/10.5281/zenodo.17864263. See DOI: https://doi.org/10.1039/d5nr04234h. There are no restrictions on data availability beyond standard privacy and ethical considerations.
