# Supplementary material for: Optical control of carrier-mediated ion transport by photoswitchable lipids
Source: Nanoscale. 2025 Dec 4;18(2):779–89. doi: 10.1039/d5nr04234h (PMC12694728; doi:10.1039/d5nr04234h)
Supplement: NR-018-D5NR04234H-s001 [file NR-018-D5NR04234H-s001.pdf]

Supplementary Information (SI) for Nanoscale.
This journal is © The Royal Society of Chemistry 2025

Supplementary Information to

Optical control of carrier-mediated ion transport by photoswitchable lipids

Juergen Pfeffermann[1,a], Rohit Yadav[1,a],
Toma Glasnov[2], Oliver Thorn-Seshold[3], Peter Pohl[1,b]

[1]Institute of Biophysics, Johannes Kepler University Linz, Linz, Austria

[2]Institute of Chemistry, University of Graz, Graz, Austria

[3]Faculty of Chemistry and Food Chemistry, Dresden University of Technology, Dresden, Germany

[a] These authors contributed equally to this work.

[b] Corresponding author. Email: peter.pohl@jku.at

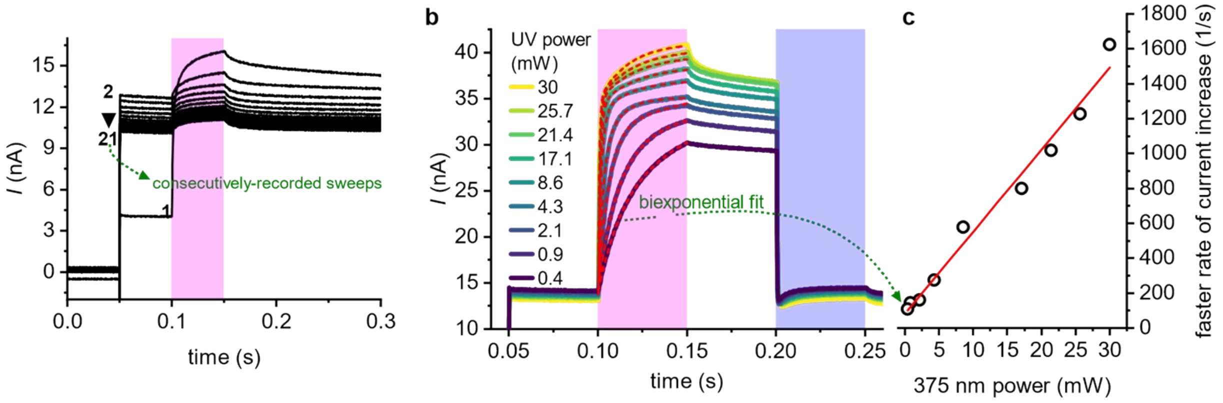

Figure S1: Time and power dependence of the UV-induced K^+-selective current increase. a, Current recordings were performed on a photoswitchable bilayer in the presence of 10 µM valinomycin on both sides (15 mM KCl, 10 mM HEPES, pH 7.4). The following voltage protocol was applied and repeated 21 times with an intersweep interval of 1 s: from 0 to 50 ms, holding potential at 0 mV; from 50 to 300 ms, voltage stepped to 130 mV; from 100 to 150 ms, the PLB was exposed to UV light. No blue light exposure occurred during the protocol. The UV-induced increase in current persists without blue light, as *cis*-azobenzene does not thermally revert to the trans isomer on this timescale (Bandara and Burdette, 2012). **b,** Irradiance-dependence of the UV light-triggered increment in *I* with valinomycin. Photoswitchable PLB with 10 µM valinomycin in 150/15 mM KCl, 10 mM HEPES pH 7.4. Recordings were conducted as in panel Figure 2b but with *V* constant at +130 mV whilst the power of the UV laser was varied in consecutive sweeps; the given values are power at the sample stage. The red-dashed lines are biexponential fits. *I* increases at a rate proportional to irradiance. **c,** Plot of the faster rate of change of *I* fitted in panel **b** over respective incident UV laser power. The red line is a fit to a linear model with offset (R^2 = 0.98).

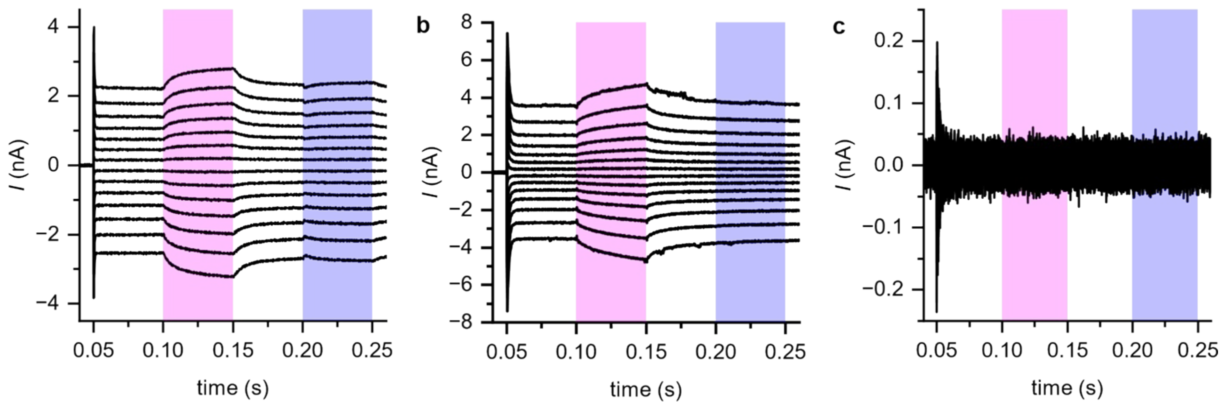

Figure S2: Lack of ion-selective current amplification in the absence of photolipids. Voltage-clamp current recordings on non-photoswitchable PLBs folded from *E. coli* PLE bathed in 15 mM KCl, 10 mM HEPES pH 7.4 containing nominally 10 µM valinomycin (panel **a**) or 10 µM CCCP (panel **b**). The records in panel **c** were made on a membrane folded from 99 wt.% *E. coli* PLE with 1 wt.% NB-lipid. Recordings were made as in Figure 2b.

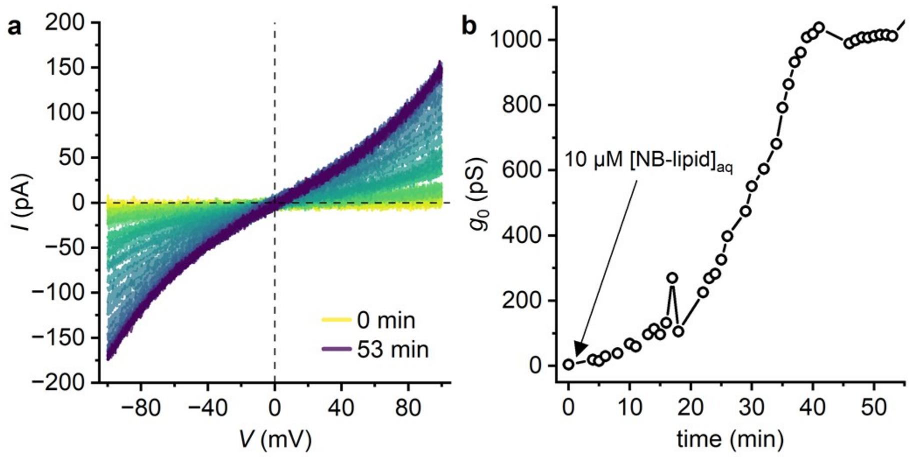

Figure S3: NB-lipid self-inserts into lipid bilayers. a, Voltage ramps measured on a vertical 135 µm-diameter photoswitchable PLB folded from 80 wt.% *E. coli* PLE and 20 wt.% OptoDArG in 15 mM KCl, 10 mM HEPES pH 7.4. 10 µM $[\text{NB-lipid}]_{aq}$ was added to both compartments (stirred continuously) immediately after the record at t = 0 min (yellow line) and further voltage ramps were recorded under continuous stirring for 53 min. Voltage protocol: 0.5 s at −100 mV, increase to 100 mV at 100 mV s^{-1}, 0.5 s at 100 mV; the constant-voltage segments are not shown. **B,** The cubic *I*–*V* curves in panel **a** were fit by the following equation: $I(V) = g_0 \cdot (1+\alpha V^2) \cdot V + o$; where $I(V)$ is the current at a particular voltage, g_0 the conductance at V = 0 mV and α is a supralinearity factor (Hannesschlaeger et al., 2019); o accounts for a small current offset. The obtained g_0 values are plot over time of record. Conductance increased gradually, indicating membrane incorporation of aqueous NB-lipid.

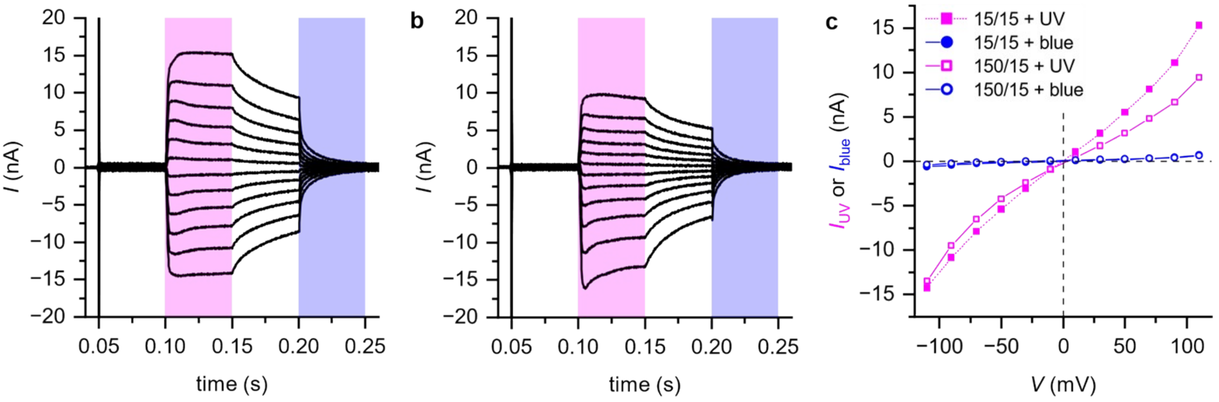

Figure S4: Relaxation and selectivity of NB-lipid induced currents. A, Voltage-clamp current recordings on a 85 µm-diameter photoswitchable PLB folded from 79 wt.% *E. coli* PLE, 20 wt.% OptoDArG and 1 wt.% NB-lipid in 15 mM KCl, 10 mM HEPES pH 7.4. Recordings were made as in Figure 2b with *V* ranging from 110 mV to −110 mV. **B,** K^+ concentration on side 1 was made 150 mM by the addition of 3 M KCl solution. Recordings were made as in panel **a**. **c,** *I*–*V* curves constructed from different intervals of the current traces in panel **a** and **b**, as described in Figure 2c.

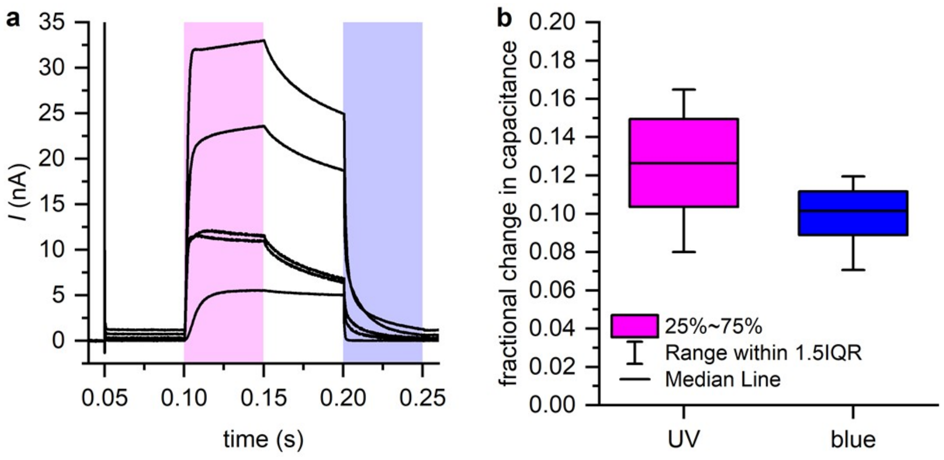

Figure S5: The increment in H^+-selective photocurrent is orders of magnitude larger than the photoinduced changes in capacitance. A, Voltage-clamp current recordings on 75 to 95 µm-diameter photoswitchable PLBs folded from 79 wt.% *E. coli* PLE, 20 wt.% OptoDArG and 1 wt.% NB-lipid in 15 mM KCl, 10 mM HEPES pH 7.4. The traces were recorded in separate measurements which were equally conducted. V = 90 mV was applied at 50 ms. Light exposure as in Figure 2b. **b,** Fractional changes in bilayer capacitance, C_m, upon UV and blue light exposure. PLBs were folded from 80 wt.% *E. coli* PLE + 20 wt.% OptoDArG (11 recordings from 3 separate experiments). Capacitance recordings were conducted as described in Bassetto *et al.* (Bassetto et al., 2024) (Figure 2a,b there). Fractional change in capacitance was calculated as $1 - C_{m,peak}/C_0$ whereby $C_{m,peak}$ denotes peak capacitance achieved within milliseconds after light exposure and C_0 capacitance immediately before light exposure.

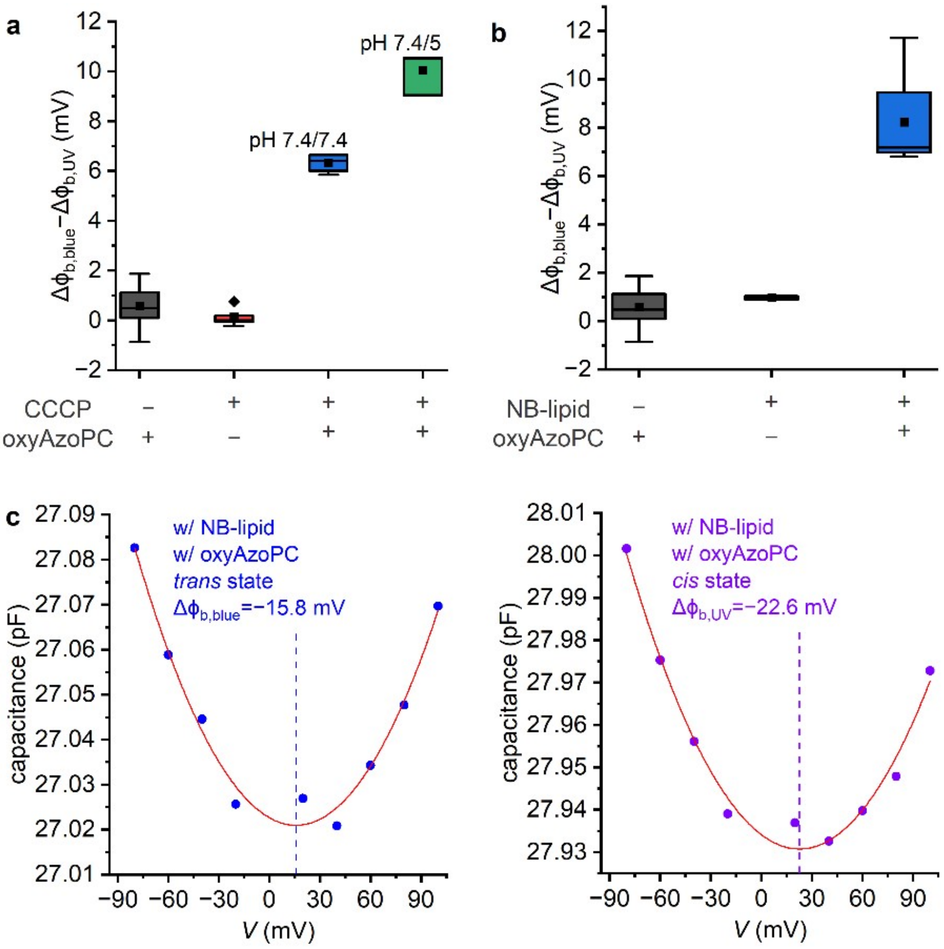

Figure S6: Azobenzene moieties offer more protonophore binding sites in the *cis* than in the *trans* state. A–b, Boundary potential measurements on PLBs in the presence of CCCP (**a**) or NB-lipid (**b**). The boundary potential difference $\Delta\phi_{b,blue} - \Delta\phi_{b,UV}$ corresponds to the voltage shift required to minimize the membrane capacitance following photoisomerization of OxyAzoPC between *trans* (blue light) and *cis* (UV light) states. PLBs were folded asymmetrically, with the leaflet facing compartment 1 composed of 80 wt.% *E. coli* polar lipid extract (PLE) and 20 wt.% of the phosphatidylcholine photolipid OxyAzoPC (**a**), or 79 wt.% PLE, 20 wt.% OxyAzoPC, and 1 wt.% NB-lipid (**b**). The opposing leaflet (adjacent to compartment 2) was composed of pure PLE in all experiments. OxyAzoPC was selected because its slow flip–flop kinetics allow confinement to a single leaflet, in contrast to the diacylglycerol OptoDArG. In control conditions, OxyAzoPC was omitted (denoted as "−" in the legend) and substituted with PLE. All experiments were performed in 15 mM KCl, 10 mM HEPES, pH 7.4. In the absence of CCCP or NB-lipid, photoisomerization of OxyAzoPC alone did not produce a significant $\Delta\phi_b$ shift. Likewise, CCCP or NB-lipid in the absence of photolipid did not induce light-dependent changes. However, in the presence of both OxyAzoPC and 2 μM CCCP, UV light induced a shift in $\Delta\phi_b$ by ≈6–7 mV (**a**), indicating increased adsorption of negatively charged $CCCP^-$ to the *cis*-OxyAzoPC-containing leaflet. In the *trans* state, this effect was modest. Lowering the pH to 5 on the photolipid-free side (to promote CCCP neutralization) further enhanced the $\Delta\phi_b$ difference. When CCCP was replaced with NB-lipid (**b**), a similar $\Delta\phi_b$ shift upon UV illumination was observed only in the presence of OxyAzoPC. Notably, the sign of the shift was the same as with CCCP, even though NB-lipid carries positive charge. This suggests that the dominant

contribution arises from changes in $\Delta\phi_d$ rather than $\Delta\phi_s$, such that the total boundary potential difference $\Delta\phi_b = \Delta\phi_d + \Delta\phi_s$ remains negative. **C**, Representative capacitance–voltage curves recorded from a PLB composed of 79 wt.% PLE, 20 wt.% OxyAzoPC, and 1 wt.% NB-lipid on side 1, and pure PLE on side 2. The voltage at which the membrane capacitance reaches a minimum corresponds in magnitude (but opposite in sign) to $\Delta\phi_b$ and was determined by quadratic fitting. Prior blue-light illumination (*trans* state) yielded a different minimum than UV-light exposure (*cis* state), with a shift of ≈6 mV. This confirms that photoisomerization modulates the electrostatic properties of the membrane via changes in the interaction of charged carriers with azobenzene moieties.

References for the Supplementary Information

Bandara, H.D., and S.C. Burdette. 2012. Photoisomerization in different classes of azobenzene. *Chem. Soc. Rev.* 41:1809-1825.

Bassetto, C.A.Z., J. Pfeffermann, R. Yadav, S. Strassgschwandtner, T. Glasnov, F. Bezanilla, and P. Pohl. 2024. Photolipid excitation triggers depolarizing optocapacitive currents and action potentials. *Nat. Commun.* 15:1139.

Hannesschlaeger, C., A. Horner, and P. Pohl. 2019. Intrinsic Membrane Permeability to Small Molecules. *Chem. Rev.* 119:5922-5953.
